# The Better to Eat You With: Bite Force in the Naked Mole-Rat (*Heterocephalus glaber*) Is Stronger Than Predicted Based on Body Size

**DOI:** 10.3389/fnint.2019.00070

**Published:** 2019-12-04

**Authors:** Natalee J. Hite, Cody Germain, Blake W. Cain, Mason Sheldon, Sai Saketh Nandan Perala, Diana K. Sarko

**Affiliations:** ^1^Department of Physiology, School of Medicine, Southern Illinois University, Carbondale, IL, United States; ^2^Southern Illinois University, Carbondale, IL, United States; ^3^School of Medicine, Southern Illinois University, Carbondale, IL, United States; ^4^Department of Electrical and Computer Engineering, Southern Illinois University, Carbondale, IL, United States; ^5^Department of Anatomy, School of Medicine, Southern Illinois University, Carbondale, IL, United States

**Keywords:** naked mole-rat, incisor, bite force, bite force quotient, bite frequency, bite latency, piezo-resistive sensor, eusocial

## Abstract

Naked mole-rats (*Heterocephalus glaber*) are subterranean rodents that utilize their incisors for feeding, chisel-tooth digging of complex tunnel systems, social interactions, and defense in their eusocial colony structure. Previous studies have shown that naked mole-rats have morphological and anatomical adaptations that predict strong bite forces, namely, skulls that are relatively tall and wide, in addition to impressive masticatory musculature. However, no studies to date have directly measured bite force in this species or analyzed the relationship between bite force and social caste. In the current study, we assessed adult naked mole-rat maximum bite force in relation to body mass, in addition to considering each animal’s position within the eusocial hierarchy (i.e., dominant versus subordinate). Each animal was permitted to freely interact with a piezo-resistive bite force sensor. Our results showed that bite force was correlated with body mass in subordinate but not in dominant naked mole-rats, and that subordinate animals exhibited a shorter latency in producing their first bite. Maximum bite force was significantly influenced by caste. In comparing bite force with available data from previous studies across 82 additional mammalian species, subordinate naked mole-rats exhibited a bite force that was 65% higher than predicted for their body size, comparable to Tasmanian devils and exceeding bite force values for all of the carnivorans included for comparison. These results supported the hypothesis that the naked mole-rat’s bite force would exceed predictions based on body size due to the behavioral importance and specialization of the naked mole-rat incisors. This study provides insight into the differences in bite force across species, and the significant role that social and ecological factors might play in the evolutionary relationship between bite force performance and underlying anatomical structures.

## Introduction

The maximum bite force across different taxa varies according to a wide range of factors including ecological niche, diet, and behavioral use of dentition. Anatomical characteristics such as body mass have proven to be significant predictors of bite force (Wroe et al., 2005; Anderson et al., 2008; Freeman and Lemen, 2008a; Marshall et al., 2012). In addition, the size of masticatory muscles for a particular species and related behavioral demands—e.g., attack and acquisition of prey, excavating tubers, digging underground tunnel systems—influence and co-vary with the strength of biting capabilities (Wroe et al., 2005; Christiansen and Wroe, 2007; Freeman and Lemen, 2008b; Becerra et al., 2014). Feeding habits in particular show a strong relationship with bite force capabilities (Wroe et al., 2005; Christiansen and Wroe, 2007; Maestri et al., 2016). Strong bite force estimates based on cranial morphology have been shown for predators that rely on the ability to effectively incapacitate and dismember prey (Christiansen and Wroe, 2007). In contrast, low bite force estimates were found for insectivores relying on a diet composed of prey that are less difficult to overpower and to consume (Aguirre et al., 2002, 2003; Wroe et al., 2005). Relatively high bite force quotients (BFQs) (comparisons to predicted bite forces across a range of taxa with different body masses based on linear regression analyses) have also been attributed to osteophages, tasked with breaking down bone material, as well as to herbivores that predominantly ingest geophytes and other hard vegetables (Herrel et al., 2002; Erickson et al., 2003; Herrel and O’Reilly, 2005; Wroe et al., 2005; Lappin et al., 2017).

**FIGURE 1:**
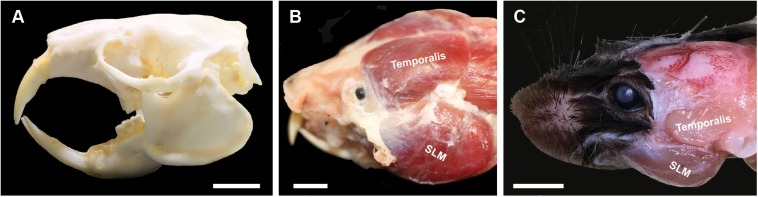
Naked mole-rat skull **(A)** and masticatory musculature **(B)** compared to that of a mouse **(C)**. **(A)** The naked mole-rat skull is characterized by large cranial width and head height, factors predictive of stronger bite forces (McIntosh and Cox, 2016). **(B)** Naked mole-rats also have large temporalis and masseter muscles, associated with strong jaw closure (biting), with the dorsal attachment site of the temporalis muscle extending toward the midline. **(C)** By comparison, the mouse has much smaller masticatory muscles, with the dorsal attachment of the temporalis muscle limited to a far lateral extent of the skull. Masseter nomenclature follows Becerra et al. (2014) for the *m. masseter lateralis, pars superficial* (superficial lateral masseter). SLM = superficial lateral masseter. Scale bar = 5 mm.

Naked mole-rats (**Heterocephalus glaber**) are a subterranean species that uses their continuously erupting incisors to dig tunnels, defend their colony, show dominance among conspecifics, and consume foods ranging from vegetables to geophytes and the bones of ungulates found near and within their tunnels (Brett, 1991). Naked mole-rats exhibit skull morphological characteristics associated with strong bite forces (Figure 1A), including large head height and cranial width (McIntosh and Cox, 2016). Previous studies have shown that naked mole-rats also have large musculature of the head and neck that facilitates feeding, digging, and social behaviors that rely on use of the incisors (Cox and Faulkes, 2014; McIntosh and Cox, 2016; Cain et al., 2019). The musculature of the jaw is responsible for approximately 25% of their total body mass (Sherman et al., 1992). The size of the muscles of mastication, in particular the masseter and temporalis muscles as seen in Figure 1B, are large compared to other species such as mice (Figure 1C). The enlarged temporalis muscles of naked mole-rats extend far medially compared to other species, meeting at the midline (Figures 1B,C). Digital dissection using microCT techniques demonstrated that the total mass of masticatory muscles in naked mole-rats was 75% of what is seen in rats, despite rats having approximately five times the body mass (Cox and Jeffery, 2011; Cox and Faulkes, 2014). Although skull measures and large muscles of mastication predict a strong bite force for naked mole-rats, and studies of other African mole-rat species have demonstrated strong bite forces (Fukomys; Van Daele et al., 2008), bite force has not been directly measured in naked mole-rats to date.

Beyond any direct bite force assessment in naked mole-rats, the influence of hierarchical social status on bite force has not been explored. As a eusocial species, naked mole-rats assume various roles within a colony. These roles range from dominant (e.g., queen, breeder) to subordinate (e.g., forager, defender, caretaker) (Sherman et al., 1992; Faulkes and Bennett, 2001). Hierarchical status is related to differences in body mass (Sherman et al., 1992) and might also be associated with differing reliance on masticatory strength used to accomplish the tasks demanded by each role such as colony defense or caring for pups (Sherman et al., 1991; Anderson et al., 2008). In the current study, we utilized piezo-resistive force sensors paired with a Raspberry Pi system and customized software to directly measure bite force from the incisors in freely behaving naked mole-rats. We then analyzed maximum bite force, bite frequency, and bite latency in relation to body mass, sex, and caste, and compared naked mole-rat maximum bite force with that of other mammalian taxa.

## Materials and Methods

### Animals

Nineteen adult (≥1 year old) naked mole-rats [*H. glaber* (Rüppell, 1842), RRID: NCBITaxon 10181], including 10 subordinate animals (five males and five females) and nine dominant animals (five males and four females), were used in this study (Table 1). The nine dominant animals consisted of all colony founders. Founders were the naked mole-rats used to initially establish our two separate laboratory colonies, and subsequently populate each respective colony; therefore, these were also the oldest colony members. The subordinate naked mole-rats were offspring of the founding group members. Body masses for the naked mole-rats included in the present study ranged from 32.4 to 94.1 g (Table 1; Braintree Scientific compact portable scale model CB 1001 with 0.1 g precision). Naked mole-rats were maintained in two separate laboratory breeding colonies, each with one queen. They were housed at ambient temperatures of approximately 27.8–30°C and at least 40% relative humidity, with free access to food. For complete housing details, see Artwohl et al. (2002). All aspects of this research complied with our protocol approved by the Institutional Animal Care and Use Committee at Southern Illinois University, Carbondale, IL, United States, and were in accordance with the National Institutes of Health Guide for the Care and Use of Laboratory Animals (NIH Publication Nos. 8023 and 1978).

**TABLE 1:** Descriptive statistics (values shown are mean ± SEM) and range for body mass, maximum bite force, bite frequency (inclusive and stringent analyses), and bite latency in naked mole-rats separated by caste.

	**Subordinate**	**Dominant**
**Animals**	*n* = 10	*n* = 9
	Five male, five female	Five male, four female (two queens)
**Age (yrs)**	1.68 ± 0.38	3.74 ± 0.21
**Body mass (g)**	56.05 ± 12.84	74.26 ± 10.68
Range	32.4–76.6	54–94.1
**Maximum bite force (N)**	21.07 ± 8.89	19.82 ± 4.68
Range	7.74–35.95	13.88–28.25
**Bite frequency (# bites/min)**		
Inclusive criteria	4.29 ± 1.70	3.39 ± 0.92
Range	2.05–7.75	2.07–5.15
Stringent criteria	1.37 ± 0.54	0.97 ± 0.31
Range	0.51–2.39	0.51–1.54
**Bite latency (min)**	1.44 ± 1.12	2.48 ± 1.66
Range	0.40–3.58	0.81–6.41

*g = grams, min = minutes, N = newton, and yrs = years.*

### Force Sensor Preparation and Calibration

We utilized FlexiForce type A201 force sensors (111N sensors; Tekscan; Boston, MA, United States) to assess naked mole-rat bite force. Per the manufacturer, each sensor has the following typical performance: linearity (error) of <±3% of full scale, repeatability of <± 2.5%, hysteresis of <4.5% of full scale, drift of <5%/logarithmic time, response time <5μsec, and an operating temperature range of −0 to 60°C. A protective coating of Plasti-Dip (Plasti-Dip International; Blaine, MN, United States) was added to prolong the life of the sensor and to maintain its responsiveness for multiple bite interactions. Plasti-Dip was applied in two coats to the distal 50 mm length of the force sensor. Each coat was given 48 h to completely cure before the coated force sensor was calibrated and utilized in experimental sessions. Each Plasti-Dipped bite force sensor was approximately 1.5 mm thick, 15 mm long, and 15 mm wide (see the inset in Supplementary Figure S1C).

In order to create calibration force curves, force sensors were conditioned following the manufacturer’s recommended parameters. Conditioning consisted of applying 110% of maximum load to the sensor until voltage response stabilized (approximately 3 s), repeated five times. A universal testing machine (UTM), specifically the Material Testing Systems (MTS) Insight 30SL Model Number 820.030-SL (MTS Systems Corporation, Eden Prairie, MN, United States), was used to calibrate our sensors. To maintain consistent force application and to achieve precise alignment, custom aluminum fixtures matching the average footprint of naked mole-rat upper and lower incisors were fabricated by the machine shop at Southern Illinois University. Setup of the UTM followed the manufacturer’s guidelines, and the force sensor was placed between the fabricated fixture interfaces. After sensor conditioning, known forces were applied to the sensor via the UTM, and the force sensor responses were recorded. A voltage per newton (V/N) curve was then directly created from the response data (Supplementary Figure S1A). The V/N ratio changes in a manner that is dependent on the input voltage resistance of the force sensor. Once the V/N ratio was calculated, bite forces measured by the probe was then converted from voltage to newton. Calibrated force sensors were paired with a Raspberry Pi Model B (Adafruit; New York City, NY, United States) using customized Python (version 3.0) software for bite force data acquisition (see Supplementary Figure S1C for detailed circuit connections).

### Bite Force Data Collection and Analysis

Each animal was placed in a chamber similar to its permanent housing chambers and was allowed to acclimate for several minutes. Next, the force sensor was inserted through a small opening at the blocked terminal end of a PVC tunnel tube to allow the animal to interact freely with the force sensor. Slightly agitating the force sensor to attract the animal’s attention generally proved effective at eliciting biting behaviors, and naked mole-rats were motivated to bite when the opening of a tunnel was blocked. Bite force measurements were then recorded for approximately 20 min after which the animal was returned to its permanent housing chamber. Five trials of approximately 20 min each were run for each animal.

The force sensor raw voltage data were sampled at 128 Hz (Supplementary Figure S1B). Data for each force sensor probe was adjusted by first subtracting the baseline “noise” voltage readings from the force sensor output, which were then converted from raw voltage output to newton values using the conversion factor for the specific probe utilized during behavioral testing based on the calibration curve for that probe, as described above regarding the V/N ration. The maximum bite force was extracted from each experimental session (trial) for five total trials per animal. The largest bite force across all five trials per animal was used for analysis of maximum bite force (Table 1). For comparisons with other rodent species and across mammalian orders, data were log transformed to normalize the distribution of the sample values (Table 2).

**TABLE 2:** Descriptive statistics (maximum bite force, body mass, residuals, and bite force quotients) across mammalian species, including data from the present study for naked mole-rats.

**Species**	**BF (N)**	**BM (g)**	**MV or C**	**Tooth**	**Residuals^1^: Rodentia**	**Residuals^2^: Rodentia**	**Residuals^1^: Mammalia**	**Residuals^2^: Mammalia**	**BFQ_1_**	**BFQ_2_**
**Rodentia**										
*Heterocephalus glaber* (naked mole-rat) castes combined	20.48^a^	64.67^a^	MV	I		0.0930		0.1814		152
*Heterocephalus glaber* (naked mole-rat) dominant only	19.82^a^	74.26^a^	MV	I	0.0273		0.1133		130	
*Heterocephalus glaber* (naked mole-rat) subordinate only	21.07^a^	56.05^a^	MV	I	0.1266		0.2169		165	
*Chinchilla laniger* (long-tailed chinchilla)	23.5^f^	639^f^	MV	I	–0.3943	–0.3923	–0.3385	–0.3367	46	46
*Ctenomys australis* (sand dune tuco-tuco)	68.7^f^	315^f^	MV	I	0.2368	0.2391	0.3026	0.3046	201	202
*Dipodomys ordii* (Ord’s kangaroo rat)	13.98^d^	63^d^	MV	I	–0.0789	–0.0758	0.0097	0.0124	102	103
*Fukomys micklemi* (African mole-rat)	41^c^	89^c^	MV	I	0.3077	0.3107	0.3914	0.3940	246	248
*Fukomys whytei* (African mole-rat)	31^c^	78^c^	MV	I	0.2171	0.2201	0.3027	0.3053	201	202
*Geomys bursarius* (plains pocket gopher)	50.61^d^	153^d^	MV	I	0.2726	0.2753	0.3487	0.3510	223	224
*Microtus ochrogaster* (prairie vole)	12.88^d^	34^d^	MV	I	0.0295	0.0330	0.1269	0.1298	134	135
*Mus musculus* (Gough Island mouse)	5.36^b^	28.60^b^	MV	I	–0.3109	–0.3073	–0.2110	–0.2080	62	62
*Mus musculus* (strain: Watkins Star Line B)	3.92^b^	19.06^b^	MV	I	–0.3520	–0.3482	–0.2464	–0.2432	57	57
*Neotoma floridana* (eastern woodrat)	30.26^d^	321^d^	MV	I	–0.1237	–0.1214	–0.0582	–0.0561	87	88
*Octodon degus* (common degus)	21.9^f^	206^f^	MV	I	–0.1606	–0.1581	–0.0888	–0.0865	82	82
*Onychomys leucogaster* (grasshopper mouse)	24.7^e^	50^e^	MV	I	0.2222	0.2255	0.3142	0.3169	206	207
*Perognathus flavescens* (plains pocket mouse)	4.64^d^	6.5^d^	MV	I	–0.0276	–0.0233	0.0933	0.0969	124	125
*Peromyscus leucopus* (white-footed mouse)	10^d^	23^d^	MV	I	0.0108	0.0145	0.1138	0.1169	130	131
*Peromyscus maniculatus* (deer mouse)	12.9^e^	21^e^	MV	I	0.1427	0.1464	0.2469	0.2500	177	178
*Rattus norvegicus* (Norway/common rat)	47^g^	555^g^	MV	I	–0.0603	–0.0583	–0.0026	–0.0007	99	100
*Reithrodontomys megalotis* (western harvest mouse)	7.67^d^	12^d^	MV	I	0.0575	0.0615	0.1703	0.1736	148	149
*Sciurus niger* (fox squirrel)	72.95^d^	588^d^	MV	I	0.1171	0.1191	0.1740	0.1759	149	150
*Sigmodon hispidus* (hispid cotton rat)	19.87^d^	105^d^	MV	I	–0.0455	–0.0426	0.0359	0.0384	109	109
*Spermophilus tridecemlineatus* (13-lined ground squirrel)	21.05^d^	144^d^	MV	I	–0.0942	–0.0915	–0.0173	–0.0149	96	97
*Zapus hudsonius* (meadow jumping mouse)	7.63^d^	24.5^d^	MV	I	–0.1214	–0.1178	–0.0193	–0.0163	96	96
**Didelphimorphia**										
*Didelphis virginiana* (opossum)	442^h^	5000^h^	MV	M			0.42629968	0.4273	267	267
*Monodelphis domestica* (gray short-tailed opossum)	21^h^	90^h^	MV	M			0.09811479	0.1007	125	126
**Carnivora**										
*Acinonyx jubatus* (Felidae) cheetah	472^i^	29500^i^	C	Ca			0.0152025	0.0155	104	104
*Alopex lagopus* (Canidae) Arctic fox	178^i^	8200^i^	C	Ca			–0.091228	–0.0905	81	81
*Canis alpinus* (Canidae) Dhole, wild dog	314^i^	16500^i^	C	Ca			–0.0179014	–0.0174	96	96
*Canis aureus* (Canidae) golden jackal	165^i^	7700^i^	C	Ca			–0.1085817	–0.1078	78	78
*Canis domesticus* (Labrador retriever)	732^j^	2864^j^	MV	Ca			0.20160883	0.2019	159	159
*Canis latrans* (Canidae) coyote	275^i^	19800^i^	C	Ca			–0.1206555	–0.1202	76	76
*Canis lupus* dingo (Canidae) dingo	313^i^	17500^i^	C	Ca			–0.0338603	–0.0334	92	93
*Canis lupus* hallstromi (Canidae) New Guinea singing dog	235^i^	12300^i^	C	Ca			–0.071005	–0.0704	85	85
*Canis lupus* lupus (Canidae) common wolf	593^i^	34700^i^	C	Ca			0.07410479	0.0743	119	119
*Crocuta crocuta* (Hyaenidae) spotted hyena	1569^i^	69100^i^	C	Ca			0.01862516	0.0186	104	104
*Dasyurus maculatus* (Dasyuridae) spotted-tail quoll	153^i^	3000^i^	C	Ca			0.09208918	0.0933	124	124
*Dasyurus viverrinus* (Dasyuridae) eastern quoll	65^i^	870^i^	C	Ca			0.02690553	0.0286	106	107
*Felis concolor* (Felidae) cougar	472^i^	34500^i^	C	Ca			–0.0235762	–0.0234	95	95
*Felis sylvestris* (Felidae) wildcat	56^i^	2800^i^	C	Ca			–0.3273262	–0.3261	47	47
*Felis yagouaroundi* (Felidae) jaguarundi/eyra	127^i^	7100^i^	C	Ca			–0.2021689	–0.2013	63	63
*Genetta tigrina* (Viverridae) Cape genet	73^i^	6200^i^	C	Ca			–0.4090781	–0.4082	39	39
*Hyaena hyaena* (Hyaenidae) striped hyena	545^i^	40800^i^	C	Ca			–0.002663	–0.0025	99	99
*Lycaon pictus* (Canidae) African hunting/painted dog	428^i^	18900^i^	C	Ca			0.0829776	0.0834	121	121
*Lynx rufus* (Felidae) bobcat	98^i^	2900^i^	C	Ca			–0.0929795	–0.0918	81	81
*Meles meles* (Mustelidae) badger	244^i^	11400^i^	C	Ca			–0.035863	–0.0352	92	92
*Neofelis nebulosa* (Felidae) clouded leopard	595^i^	34400^i^	C	Ca			0.07771769	0.0779	120	120
*Panthera leo* (Felidae) lion	1768^i^	294600^i^	C	Ca			0.01877883	0.0182	104	104
*Panthera onca* (Felidae) jaguar	1014^i^	83200^i^	C	Ca			0.09049162	0.0904	123	123
*Panthera pardus* (Felidae) leopard	467^i^	43100^i^	C	Ca			–0.0833255	–0.0832	83	83
*Panthera tigris* (Felidae) tiger	1525^i^	186900^i^	C	Ca			0.06727106	0.0668	117	117
*Proteles cristatus* (Hyaenidae) aardwolf	151^i^	9300^i^	C	Ca			–0.1938489	–0.1931	64	64
*Sarcophilus harrisii* (Dasyuridae) Tasmanian devil	418^i^	12000^i^	C	Ca			0.18521922	0.1858	153	153
*Urocyon cinereoargenteus* (Canidae) gray fox	114^i^	5300^i^	C	Ca			–0.1766497	–0.1757	67	67
*Ursus americanus* (Ursidae) American black bear	541^i^	105200^i^	C	Ca			–0.2404583	–0.2407	57	57
*Ursus arctos* (Ursidae) brown bear	751^i^	128800^i^	C	Ca			–0.1481451	–0.1484	71	71
*Ursus thibetanus* (Ursidae) Asian black bear	312^i^	77200^i^	C	Ca			–0.4028536	–0.4030	40	40
*Vulpes vulpes* (Canidae) red fox	164^i^	8100^i^	C	Ca			–0.1237652	–0.1230	75	75
**Chiroptera**										
*Cynopterus brachyotis* (lesser short-nosed fruit bat)	12^k^	44^k^	MV	Ca			0.03232018	0.0351	108	108
*Eidolon helvum* (straw-colored fruit bat)	92^k^	272^k^	MV	M			0.39405996	0.3962	248	249
*Pteropus poliocephalus* (gray-headed flying fox)	117^k^	820^k^	MV	M			0.02799251	0.0297	107	107
*Pteropus vampyrus* (large/greater flying fox)	163^k^	1166^k^	MV	M			0.07088074	0.0724	118	118
*Rousettus egyptiacus* (Egyptian fruit bat)	32^k^	179^k^	MV	M			–0.1156485	–0.1134	77	77
*Artibeus jamaicensis* (Jamaican fruit bat)	19^k^	45^k^	MV	Ca			0.2263265	0.2291	168	169
*Carollia perspcillata* (Seba’s short-tailed bat)	4^k^	18^k^	MV	Ca			–0.2234219	–0.2203	60	60
*Desmodus rotundus* (common vampire bat)	9^l^	41^l^	MV	Ca			–0.0751281	–0.0723	84	85
*Eptesicus furinalis* (Argentine brown bat)	7^l^	9^l^	MV	Ca			0.19129354	0.1947	155	157
*Erophylla sezekorni* (buffy flower bat, in the leaf-nosed bat family)	3^k^	17^k^	MV	Ca			–0.3342038	−−0.3310	46	47
*Glossophaga soricina* (Pallas’s long-tongued bat)	1^l^	11^k^	MV	Ca			–0.7035062	–0.7001	20	20
*Micronycteris minuta* (white-bellied big-eared bat)	2^l^	8^l^	MV	Ca			–0.3236022	–0.3201	47	48
*Mimon crenulatum* (striped hairy-nosed bat)	7^l^	16^l^	MV	Ca			0.04878841	0.0520	112	113
*Molossus rufus* (black mastiff bat)	8^l^	29^l^	MV	Ca			–0.0405156	–0.0375	91	92
*Monophyllus redmani* (Leach’s single leaf bat/greater Antillean long-tongued bat)	1^k^	13^k^	MV	Ca			–0.7448819	–0.7416	18	18
*Myotis albescens* (silver-tipped myotis)	2^l^	5^l^	MV	Ca			–0.2071926	–0.2035	62	63
*Myotis nigricans* (black myotis)	1^l^	4^l^	MV	Ca			–0.4529548	–0.4492	35	36
*Myotis simus* (velvety myotis)	3^l^	8^l^	MV	Ca			–0.147511	–0.1440	71	72
*Noctilio leporinus* (greater bulldog/fisherman bat)	20^l^	63^l^	MV	Ca			0.16526608	0.1679	146	147
*Noctilo albiventris* (lesser bulldog bat)	12^l^	34^l^	MV	Ca			0.09617882	0.0991	125	126
*Phyllostomus elongatus* (lesser spear-nosed bat)	15^l^	35^l^	MV	Ca			0.18590925	0.1888	153	154
*Phylostomus discolor* (pale spear-nosed bat)	22^l^	37^l^	MV	Ca			0.33847724	0.3414	218	219
*Sturnira lilium* (little yellow-shouldered bat)	8^l^	20^l^	MV	Ca			0.05151258	0.0546	113	113
*Tonatia silvicola* (white-throated round-eared bat)	22^l^	27^l^	MV	Ca			0.41651593	0.4195	261	263
*Uroderma bilobatum* (tent-making bat)	10^l^	23^l^	MV	Ca			0.11380662	0.1169	130	131
**Primate**										
*Homo sapiens* (humans)	749^o^	2874^p^	MV	I			0.04659657	0.0466	111	111
*Pongo pygmaeus* (orangutan)	1712^m^	3233^n^	C	M			0.41337255	0.4134	259	259

*^1^Naked mole-rat castes separated (subordinate, dominant); ^2^Naked mole-rat castes combined (subordinate + dominant). ^*a*^Hite et al., 2019; ^*b*^Parmenter et al., 2019; ^*c*^Van Daele et al., 2008; ^*d*^Freeman and Lemen, 2008a; ^*e*^Williams et al., 2009; ^*f*^Becerra et al., 2014; ^*g*^Robins, 1977; ^*h*^Thomason et al., 1990; ^*i*^Wroe et al., 2005; ^*j*^Ellis et al., 2008; ^*k*^Dumont and Herrel, 2003; ^*l*^Aguirre et al., 2002; ^*m*^Lucas et al., 1994; ^*n*^Silva and Downing, 1995; ^*o*^Van Eijden, 1991; ^*p*^Ruff, 1994. Bite force data cited from previous studies were either directly measured values from behavioral studies or calculated values based on skull morphology parameters or other factors (categorical nomenclature follows Van Daele et al., 2008). BF = bite force, BM = body mass, BFQ = bite force quotient, C = calculated value, Ca = canine, g = grams, I = incisor, M = molar, MV = measured value, and N = newton.*

All statistical analyses were performed using IBM SPSS Statistics version 26. In order to further facilitate cross-species comparisons for taxa spanning a wide range of body masses, we also calculated BFQs by applying our linear regression analyses for Mammalia (Table 2) following similar BFQ analyses from previous studies (Wroe et al., 2005; Christiansen and Wroe, 2007). To calculate BFQ, the following equation was used for separated naked mole-rat castes (dominant and subordinate; Table 2):

$$B\,F\,Q_{1}=100\,\left(\frac{B\,F}{10^{0.5703\,\left(\log_{10}B\,M\right)+0.1096}}\right)$$

where BF is bite force and BM is body mass. For combined naked mole-rat castes (Table 2), the following equation was used:

$$B\,F\,Q_{2}=100\,\left(\frac{B\,F}{10^{0.5712\,\left(\log_{10}B\,M\right)+0.1053}}\right)$$

For bite latency and bite frequency analyses, comparisons were made using the mean values across the five trials per animal (Table 1). We used Matlab to locate the first data point that passed the force threshold (at least two standard deviations above the mean of all recorded force sensor data for that session from the start time of each trial) to qualify as a bite and to assess latency. In addition, two separate bite frequency analyses were performed using Matlab to quantify the number of individual bites that occurred per experimental session above the force threshold. The first was an inclusive bite frequency analysis in which all points above threshold were counted (Supplementary Figure S2A). The second applied more stringent criteria and required a minimum distance of 50 acquired data points between any point surpassing the threshold in order to assess separate bite events (Supplementary Figure S2B). The values acquired in Matlab for bite latency, inclusive bite frequency, and stringent bite frequency were imported into SPSS Statistics version 26 for further statistical analyses. Separate ANOVA analyses used either bite frequency, bite latency, or bite force as the dependent variable; sex and caste as fixed factors; and body mass as a covariate in testing for the main effect of caste on each dependent variable. Age was not included in analyses due to its significant correlation with body mass (Spearman correlation coefficient of 0.570, *p* = 0.011).

## Results

### Qualitative Assessment of Biting Behaviors in Naked Mole-Rats

Overarching trends observed during bite force assessments characterized naked mole-rat biting behaviors as defensive, exploratory, digging, or chewing. One such behavior was a tendency to bite the edge of the probe and to exhibit what appeared to be defensive biting behavior. Upon grasping the probe’s edge between its upper and lower incisors, the naked mole-rat would simultaneously run backward and twist its head as if attempting to tear away part of the probe (similar to the manner in which a person might twist their head while biting to tear off a piece of tough food matter such as beef jerky). A second noteworthy behavior in naked mole-rats was a tendency to gnaw on the probe in what appeared to be exploratory biting rather than defensive behavior. During exploratory biting, rather than retreating backward through its tunnel system and twisting its head, the animal kept the probe between its upper and lower incisors while continuously applying pressure to the probe and pulling on it, paired with bracing and pressing of the forelimbs and occasionally even lifting the hindlimbs off the ground. As each experimental session progressed, animals more frequently exhibited a forelimb digging motion targeting the site where the probe emerged through a slot at the tunnel’s terminal. If the probe was temporarily removed, animals would occasionally place their upper incisors within the slot of the tunnel terminal and would gnaw at the edges, keeping the upper incisors in place while scraping the lower incisors along the tunnel’s edge in a chisel-tooth digging fashion (Olivares et al., 2004; Samuels and Van Valkenburgh, 2009; McIntosh and Cox, 2016). If food was offered in place of the probe at the tunnel’s terminal, the naked mole-rat would tear off parts of the food (e.g., sweet potato) with its incisors and retreat backward through the tunnel to the larger housing chamber where it grasped the food between its forepaws to steady it during consumption. The upper incisors were used to hold the food item in place while the lower incisors were moved in such a way so as to dislodge and scoop smaller pieces of the food that could be consumed with multiple smaller bites using the molars. Naked mole-rats likely modulate bite force across these different categories of biting behaviors in order to optimize outcomes, with defensive behaviors associated with the strongest bite forces in order to effectively protect the colony.

### Naked Mole-Rat Bite Frequency and Bite Latency With Respect to Caste

**FIGURE 2:**
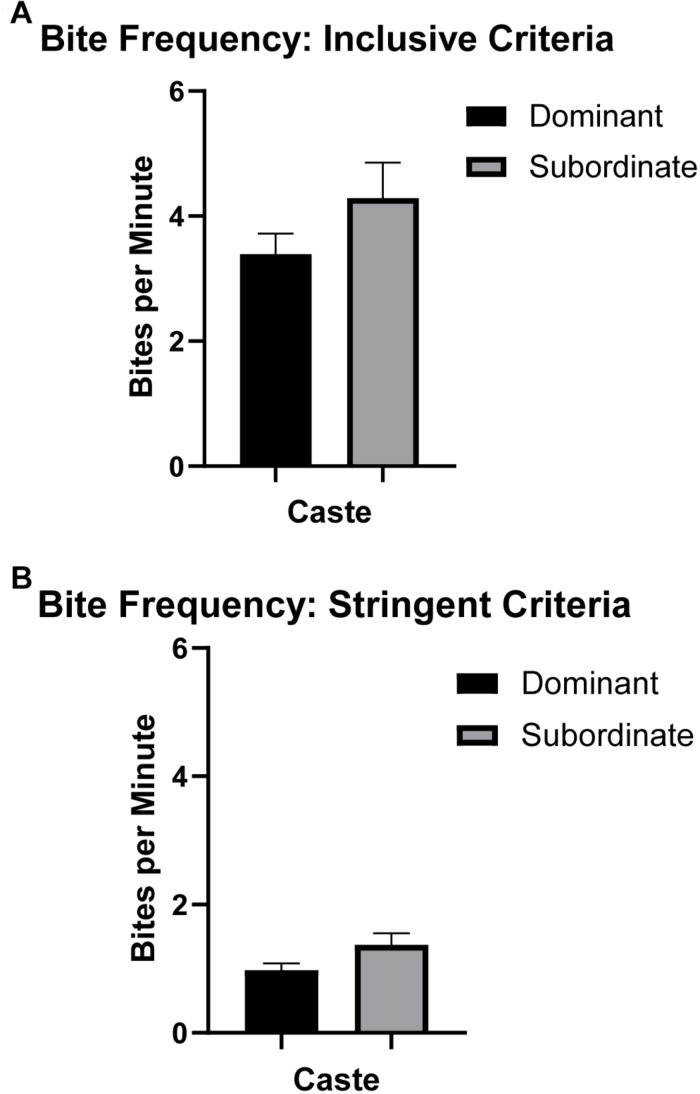
Bite frequency (mean ± SEM) for dominant (black, *n* = 9) and subordinate (gray, *n* = 10) naked mole-rats. One-way ANOVA analysis revealed no significant influence of caste (accounting for sex and body mass) on mean bite frequency in naked mole-rats. (See the section “Materials and Methods” and Supplementary Figure S2 for details regarding inclusive versus stringent criteria for bite frequency analyses.) **(A)** For inclusive bite frequency analyses where all data points above threshold were counted as bites, there were no significant differences by caste (dominant = 3.39 ± 0.92, subordinate = 4.29 ± 1.70). **(B)** For stringent bite frequency analyses classifying separate bite “events,” similarly there were no significant differences by caste (dominant = 0.97 ± 0.31, subordinate = 1.37 ± 0.54).

**FIGURE 3:**
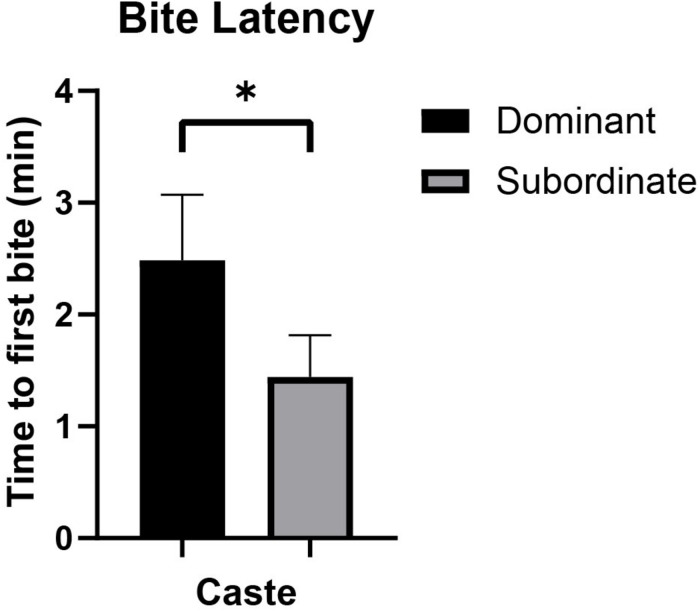
Bite latency (mean ± SEM) for dominant (black, *n* = 9) and subordinate (gray, *n* = 10) naked mole-rats. A one-way ANOVA revealed that caste significantly affected bite latency [*F*_(1,18)_ = 4.666, *p* = 0.049], accounting for body mass and sex. Mean bite latency for dominant animals was 2.48 ± 1.66 min, whereas mean bite latency for subordinate animals was 1.44 ± 1.12 min. min = minutes. ^∗^ signifies *p* < 0.05.

Bite frequency and bite latency (Table 1) were analyzed to assess differences between castes that might indicate differences in motivation to bite (e.g., high bite frequency or short bite latency potentially indicating increased motivation and/or decreased inhibition to bite). Bite frequency ANOVA analyses found no significant differences in bite frequency between dominant versus subordinate animals whether the analysis was based on more inclusive criteria [*F*_(__1_,_18__)_ = 1.431, *p* = 0.251; Figure 2A] or more stringent criteria [*F*_(__1_,_18__)_ = 2.765, *p* = 0.119; Figure 2B], adjusting for body mass and sex. (Also see Supplementary Figure S2 for an illustration of each bite frequency criterion applied to representative data.) Thus, these values can be combined across castes to yield an average bite frequency of 3.895 bites/min using inclusive criteria and 1.185 bites/min using stringent criteria. In contrast, quantification of latency to first bite showed significant effects of caste when body mass and sex were accounted for [*F*_(__1_,_18__)_ = 4.666, *p* = 0.049; Figure 3]. Dominant naked mole-rats took nearly twice as long to produce their first bite compared to subordinate animals (2.48 ± 1.66s versus 1.44 ± 1.12s, respectively; Table 1).

### Naked Mole-Rat Maximum Bite Force With Respect to Caste

**FIGURE 4:**
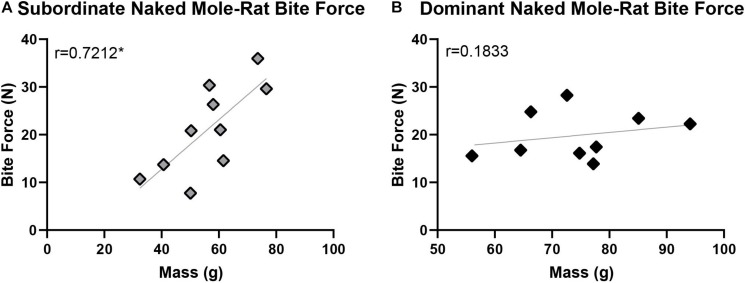
Maximum bite force was significantly correlated with body mass in subordinate naked mole-rats (**A**; *n* = 10; *r* = 0.7212, *p* = 0.019) but not in dominant naked mole-rats (**B**; *n* = 9; *r* = 0.1833, *p* = 0.637). ANOVA analysis further confirmed that maximum bite force was significantly influenced by caste, adjusting for body mass and sex [*F*_(1,18)_ = 6.212, *p* = 0.026]. Black represents dominant naked mole-rats and gray represents subordinate naked mole-rats. ^∗^ signifies *p* < 0.05.

For the naked mole-rats included in this study, body mass—shown by previous studies in other species to be a key predictor of maximum bite force—was distributed across a relatively wide range of 32.4–94.1 g (Table 1). The maximum bite forces produced also varied across a wide range of 7.74–35.95 N, with an average maximum bite force of 19.82 ± 4.68 N in dominant naked mole-rats and 21.07 ± 8.89 N in subordinate naked mole-rats (Table 1). When naked mole-rats were separated by caste, distinct differences in maximum bite force were observed with respect to body mass. Initial correlation assessments of each group showed that maximum bite force was significantly and positively correlated with body mass in subordinate animals (Spearman correlation coefficient of 0.7212, *p* = 0.019; Figure 4A), as predicted based on other species. However, in dominant animals, maximum bite force was not correlated with body mass (Spearman correlation coefficient of 0.1833, *p* = 0.637; Figure 4B). Thus, whereas bite force increased with body mass in subordinate animals, it remained similar irrespective of body mass in dominant animals. ANOVA analysis further confirmed that maximum bite force showed a significant main effect of caste [*F*_(__1_,_18__)_ = 6.212, *p* = 0.026], adjusting for body mass and sex, indicating that a naked mole-rat’s role within the eusocial hierarchy was significantly interrelated with maximum bite force when the comparison between castes was conducted on a body mass-corrected bite force measure. There was also a significant main effect of body mass on bite force [*F*_(__1_,_18__)_ = 10.800, *p* = 0.005]. Sex approached but did not reach a significant effect on bite force [*F*_(__1_,_18__)_ = 4.298, *p* = 0.057], and there was no significant interaction between caste and sex on bite force measures [*F*_(__1_,_18__)_ = 0.209, *p* = 0.654].

### Maximum Bite Force in Naked Mole-Rats Compared to Other Mammalian Species

**FIGURE 5:**
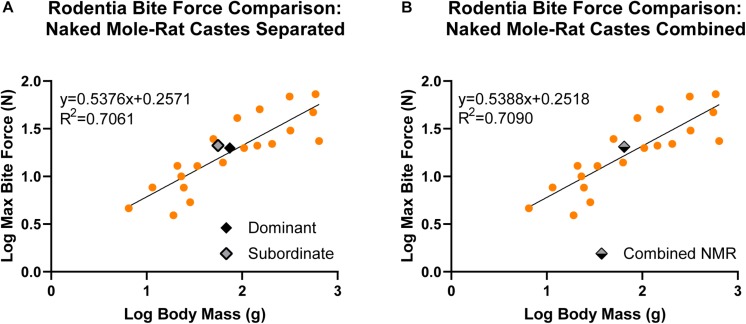
Linear regression analyses comparing maximum bite force to body mass in naked mole-rats together with 21 additional rodent species (data from other rodent species obtained from previous studies; see Table 2 for associated references). Data were log transformed to normalize the distribution of the sample values. There was a significant, positive allometric relationship between bite force and body mass across Rodentia. **(A)** When naked mole-rats were separated by caste (*y* = 0.5376*x* + 0.2571), subordinate animals (gray, *n* = 10) were above the regression line (residual = 0.1266) whereas dominant animals (black, *n* = 9) fell closer to the regression line (residual = 0.0273). **(B)** When dominant and subordinate castes were combined (gray/black; *y* = 0.5388*x* + 0.2518), naked mole-rats exhibited bite forces above the regression line (residual = 0.0930). g = grams, N = newton, and NMR = naked mole-rat.

There was a significant, positive allometric relationship between maximum bite force and body mass analyzed across naked mole-rats and 21 additional species of Rodentia whether naked mole-rat castes were separated (*y* = 0.5376*x* + 0.2571, *R*^2^ = 0.7061, *p* < 0.001; Figure 5A and Table 2) or combined (*y* = 0.5388*x* + 0.2518, *R*^2^ = 0.7090, *p* < 0.001; Figure 5B and Table 2). Compared to other rodents, when naked mole-rats were separated by caste (Figure 5A), subordinate naked mole-rats exhibited bite forces that exceeded predicted values (residual of 0.1266) whereas dominant naked mole-rats exhibited bite forces that more closely approximated predicted values (residual of 0.0273; Table 2). This equated to a predicted bite force of 15.74 N for subordinate animals compared to our directly measured average value of 21.07 N, which was 34% greater than the predicted subordinate bite force. Dominant animals were predicted to have a bite force of 18.61 N, compared to our directly measured average value of 19.82 N, which was only 6.5% greater than predicted and more closely matched the predicted dominant bite force. Combining subordinate and dominant castes to be grouped together (Figure 5B) brought the naked mole-rat residual to 0.0930 (Table 2) with a predicted bite force of 16.53 N, compared to our directly measured average value of 20.48 N for the combined castes, which was 24% greater than the predicted bite force. Thus, naked mole-rats exhibited a bite force that was stronger than predicted for their body mass compared to other rodent species, and this was primarily driven by the performance of subordinate naked mole-rats.

**FIGURE 6:**
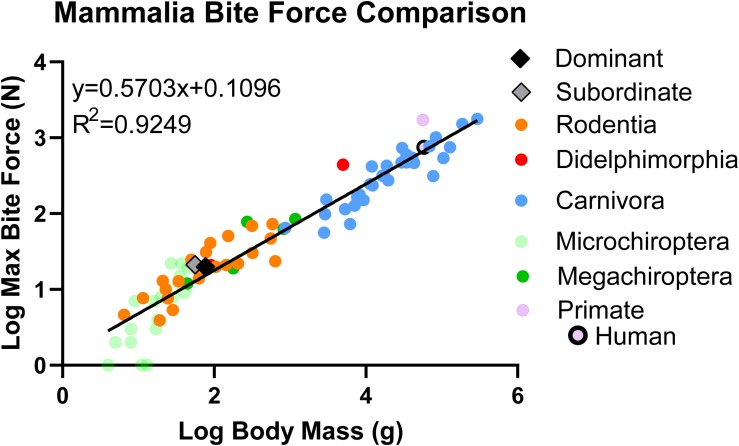
Linear regression analyses comparing bite force to body mass in naked mole-rats together with 82 additional mammalian species (data from other species obtained from previous studies; see Table 2, color-coded by mammalian order in congruence with this figure, for references). Data were log transformed to normalize the distribution of sample values. The residual for subordinate naked mole-rats (gray, *n* = 10) was 0.2169 whereas the residual for dominant naked mole-rats was 0.1133 (black, *n* = 9), compared to 0 for the common rat. This demonstrated that naked mole-rats exhibited a bite force that was much stronger than predicted for their body size.

The significant, positive relationship between bite force and body mass was also found when the Rodentia comparison was broadened to encompass additional mammalian orders for a total of 83 mammalian species, including naked mole-rats separated by caste (*y* = 0.5703*x* + 0.1096, *R*^2^ = 0.9244, *p* < 0.001; Figure 6 and Table 2). Within this scope, naked mole-rat bite force exceeded predicted values for each caste when separated (residual of 0.2169 for subordinate naked mole-rats, residual of 0.1133 for dominant naked mole-rats). The cross-order mammalian comparison generated a predicted bite force of 12.79 N for subordinate naked mole-rats (compared to our directly measured average value of 21.07 N, which was 65% greater than the predicted subordinate bite force) and 15.27 N for dominant naked mole-rats (compared to our directly measured average value of 19.82 N, which was 30% greater than the predicted dominant bite force). Combining subordinate and dominant castes (*y* = 0.5712*x* + 0.1053, *R*^2^ = 0.9252, *p* < 0.001) generated a residual of 0.1814 and a predicted bite force of 13.49 N for naked mole-rats (compared to our directly measured average value of 20.48 N for the combined castes, which was 52% greater than the predicted bite force). Overall, in comparison to a wide range of mammalian species spanning multiple orders, naked mole-rat bite force remained much stronger than predicted based on body size, particularly for subordinate animals.

Although BFQ results were similar for other mammalian species whether they were based on linear regression analyses that included naked mole-rat castes as separated (BFQ_1_, Table 2) or combined (BFQ_2_, Table 2), we included both sets of values for completeness and to enable either set of values to be applied to and compared with future studies. Species with BFQs of approximately 100 have bite forces equal to those predicted by the cross-species comparison for their body size (Wroe et al., 2005; Christiansen and Wroe, 2007). Subordinate and dominant naked mole-rats exhibited BFQs that exceeded predicted values (164.78 and 129.79, respectively) whereas grouping castes together produced a naked mole-rat BFQ of 151.74, altogether indicating that naked mole-rat bite force was much greater than predicted based on body mass in comparison to other mammals spanning a wide range of body masses and bite forces (Table 2).

## Discussion

Naked mole-rats were assessed across multiple variables including bite frequency, bite latency, and maximum bite force in order to characterize their biting behaviors in relation to body mass and caste. Other studies that have assessed mammalian bite frequency have focused on chewing behaviors rather than the defensive or exploratory bites that predominantly characterized naked mole-rat biting behaviors in our study. Chewing frequency has been shown to negatively correlate with body mass in mammals, with a ceiling effect imposed by masticatory muscle capabilities (Druzinsky, 1993; Virot et al., 2017). Mammalian species studied for chewing frequency included lions (1.43 ± 0.41 Hz), orangutans (1.21 ± 0.24 Hz), humans (1.71 ± 0.42 Hz), and a chinchilla (4.24 ± 0.59 Hz) (Virot et al., 2017). Bite frequency, as measured in the current study, does not directly compare to chewing frequency, with naked mole-rats averaging a bite frequency of approximately 1 bite/min using stringent analysis criteria (Table 1, Supplementary Figure S2B, and Figure 2B). Bite frequency was not associated with caste. In contrast to bite frequency in naked mole-rats, bite latency was significantly associated with caste. Dominant animals took nearly twice as long to initially bite the force sensor compared to subordinate animals (Table 1 and Figure 3). Very few studies have characterized mammalian bite frequency and bite latency, particularly with respect to the relationship of each of these variables with body mass or social hierarchical status. Social behaviors attributable to lower-ranking versus high-ranking animals may in part drive the influence of caste on bite latency. Subordinate animals may have a higher motivation and/or less inhibition to initiate biting behaviors, whereas dominant animals with a more established position in the colony hierarchy may exhibit less motivation and/or greater inhibition to initiate biting behaviors. Bite latency has been examined in rodents using behavioral tests of aggression which showed that shorter bite latencies were associated with increased aggression (Koolhaas et al., 2013). Latency to bite has not been previously analyzed with respect to body mass and caste, rendering it difficult to compare the relationships between these variables in naked mole-rats to those observed in other species.

The average bite force was 21.07 ± 8.89 N in subordinate naked mole-rats and 19.82 ± 4.68 N in dominant naked mole-rats (Table 1). Bite force was positively and significantly correlated with body mass in subordinate but not in dominant naked mole-rats (Figure 4). When assessed with 21 additional species of Rodentia (Figure 5 and Table 2) or expanded to 82 additional species of Mammalia (Figure 6 and Table 2), naked mole-rat bite force exceeded the values predicted based on body size, particularly for subordinate animals.

### Naked Mole-Rat Bite Force Compared to Other Mammalian Species

The maximum bite force of naked mole-rats was greater than expected based on predicted values, whether these comparisons were limited to other rodents (Figure 5) or expanded across mammalian orders (Figure 6). Subordinate and dominant naked mole-rats exhibited BFQ values of 165 and 130, respectively (Table 2), whereas a measured bite force that matched predictions based on body size would have produced BFQ values of 100 (Wroe et al., 2005; Christiansen and Wroe, 2007). For their body size, subordinate naked mole-rats outperformed all of the carnivorans included for comparison, such as American black bears (BFQ of 57), lions (BFQ of 104), and wolves (BFQ of 119), all of which require strong bite forces in order to shear the muscle and crack the bones of their prey (Table 2). Subordinate naked mole-rat bite force was comparable to the value calculated for Tasmanian devils (BFQ of 153; Table 2). Like some of the carnivorans in the Mammalia comparisons, naked mole-rats occasionally consume bone, a feat associated with large bite forces (Wroe et al., 2005; Christiansen and Wroe, 2007). Often, species with higher than predicted bite forces are also animals that hunt prey larger than themselves (Christiansen and Wroe, 2007). Naked mole-rats share some similarities with these species in that they require stronger bite forces for adversaries such as snakes that far exceed their own average body size (Sherman et al., 1991). In addition, reliance on different biting strategies also affects maximum bite force. Wroe et al. (2005) categorized distinct biting strategies for mammals, distinguishing between those that used static versus kinetic bites, the latter of which requires more force. The kinetic bites consisted of stabbing bites targeted at the neck and ones that sheared with their canines.

Naked mole-rats outperformed humans as well, with a human BFQ of 111 that closely approximated the value predicted for their body size (Table 2). If the bite force exhibited by subordinate naked mole-rats were extrapolated to the body mass of humans (2874 g; Table 2; Van Eijden, 1991), a human-sized naked mole rat would exhibit a bite force of 1110 N—48% greater than the measured human bite force. By comparison, an American black bear-sized naked mole-rat would produce a bite force of 1553 N, or 187% greater than the calculated black bear bite force; a lion-sized naked mole rat would yield a bite force of 2794 N, or 58% stronger than the lion’s calculated bite force; and a wolf-sized naked mole-rat would produce a bite force of 825 N, or 39% stronger than the wolf’s calculated bite force (Table 2; Wroe et al., 2005).

Although naked mole-rats exhibited higher BFQs than the majority of other mammalian species included for comparison in the present study, they were out-performed by certain species including orangutans (Lucas et al., 1994), several bat species (Aguirre et al., 2002; Dumont and Herrel, 2003), and opossums (Thomason et al., 1990), in addition to several rodent species (Table 2). These rodent species included other species of subterranean mole-rats, the African mole-rats (*Fukomys micklemi* and *Fukomys whytei*, Van Daele et al., 2008), as well as the plains pocket gopher (Freeman and Lemen, 2008a), the grasshopper mouse (Williams et al., 2009), and the sand dune tuco-tuco (*Ctenomys australis*, Becerra et al., 2014). *C. australis* is a subterranean species that relies on both chisel- and scratch-tooth digging (Becerra et al., 2013). Socially, *C. australis* is highly aggressive, territorial, and solitary (Becerra et al., 2013)—traits shared with the ground-dwelling, carnivorous grasshopper mouse (Satoh and Iwaku, 2006; Williams et al., 2009) as well as with the fossorial pocket gopher (Freeman and Lemen, 2008a). Naked mole-rats, by comparison, are subterranean chisel-tooth diggers that are eusocial and can be aggressive as needed for colony defense or intraspecific competition. Chisel-tooth diggers are characterized by well-developed head and neck musculature utilized in biting, as well as adaptations in skull morphology such as large zygomatic arches and temporal fosse that facilitate usage of the incisors as chisel-like tools (Samuels and Van Valkenburgh, 2009). McIntosh and Cox (2016) classified chisel-tooth diggers as more capable of producing stronger bites than their scratch-digging counterparts based on dental and skull morphology related to increased behavioral reliance on and utilization of masticatory musculature.

Bite force can also be influenced by masticatory muscle variations, such as the microstructural composition and relative size of the temporalis and masseter muscles (Olivares et al., 2004), as well as differences in craniofacial morphology and gape (McIntosh and Cox, 2016). Such variations, together with divergent evolutionary pressures, likely contribute to the discrepancies in predicted versus actual bite force, and differing BFQ values (Table 2). Maximum gape is dictated by skull and muscular parameters in that gape increases with increased resting and stretch length of masticatory muscles, increased size of the temporalis muscles, increased jaw length, increased condyle length, and decreased condyle height (Satoh and Iwaku, 2006; Cox and Faulkes, 2014). Variations in gape, in turn, strongly influence bite force such that bite force negatively scales with gape angle (Dumont and Herrel, 2003; Santana, 2016). Bite force also varies according to the tooth being assessed, with higher bite forces associated with molars compared to incisors (Dumont and Herrel, 2003). Due to these factors, differences observed in our cross-mammalian comparison were likely affected by, and must be considered within the context of, the tooth being assessed for bite force (canine, molar, or incisor) and associated gape differences (Table 2).

### Naked Mole-Rat Bite Force and Body Mass: Relationship to Behavioral Roles Within the Eusocial Colony Structure

Maximum bite force was correlated with body mass for subordinate naked mole-rats (Figure 4A), as predicted based on the relationship between body mass and bite force demonstrated in a wide range of other taxa (Thomason et al., 1990; Lucas et al., 1994; Aguirre et al., 2002; Dumont and Herrel, 2003; Thompson et al., 2003; Wroe et al., 2005; Freeman and Lemen, 2008a; Ellis et al., 2009; Williams et al., 2009; Becerra et al., 2014). However, in dominant naked mole-rats, bite force remained fairly constant and was not correlated with body mass (Figure 4B). Further analysis confirmed that bite force was significantly influenced by caste. However, it should be noted that the dominant naked mole-rats in our sample are also inherently larger and older animals, and it may be the case that the correlation with bite force weakens at higher body masses rather than differences between dominant and subordinate naked mole-rats being due exclusively to caste-specific influences. When dominant and subordinate groups were combined, naked mole-rats exhibited a maximum bite force that exceeded the predicted value based on their body mass. Separating these groups by caste demonstrated that this difference was primarily driven by subordinate naked mole-rats, such that these animals exhibited bite forces well beyond those predicted for their body mass whereas dominant naked mole-rats more closely adhered to predicted values.

The eusocial colony structure of naked mole-rats is related to behavioral and body size differences between castes, as well as differing role requirements for animals within each caste. Within the present study, the dominant group was composed solely of founding members of each naked mole-rat colony. Previous studies have shown significant differences in body mass between larger naked mole-rats that established a colony (i.e., founders) versus smaller animals that were subsequently birthed into the colony, in addition to body mass fluctuations in response to changes in or disruption of the eusocial hierarchy (O’Riain and Jarvis, 1998). The first litter of a nascent colony responded most strongly to social structure changes within the colony, such as death of a male breeder, with distinct increases in body mass. O’Riain and Jarvis (1998) were also able to show that the subsequent litters (referred to in the current study as subordinates) maintained smaller body sizes unless particular animals diverged to defensive or breeding roles within the colony, at which point these animals greatly increased in body mass as a direct result of assuming a new role in the social hierarchy. When naked mole-rats were classified as breeders (more dominant) and non-breeders (more subordinate), behaviors involving utilization of the incisors such as nest building, digging, transporting food, and defense were associated with differences in body mass (Sherman et al., 1991).

As opposed to dominant naked mole-rats, subordinate animals exhibited a significant correlation between body mass and bite force. This may be related to the broad spectrum of behavioral roles and associated differences in body mass observed in subordinate naked mole-rats. Larger non-breeding subordinate animals engage in chisel-tooth digging required for excavating new tunnels. The largest of the subordinate naked mole-rats have been observed to be “volcanoers,” animals that hold positions at the openings of underground tunnels to the surface in order to kick out dirt (which takes on the outward appearance of sandy volcanoes). This is by necessity a defensive behavioral role due to the proximity to the surface rendering these animals vulnerable to predators. The large body size of these animals may also be related to the strong musculature needed to move large volumes of dirt, as is required for tunnel “volcanoeing” (Hamilton, 1928). The larger subordinate animals perform tasks that require more forceful utilization of masticatory musculature than their smaller counterparts that tend to focus on nesting behaviors, thereby aligning with the positive significant correlation of body mass with bite force. As described above, differences in gape between smaller versus larger animals may also have contributed to the bite force differences observed. However, our bite force sensors were small (1.5 mm thick) relative to the head size of naked mole-rats (see the inset in Supplementary Figure S1C) and should have produced minimal differences in gape across the naked mole-rats examined. Nonetheless, the influence of gape angle on bite force would be more pronounced in subordinate naked mole-rats, with the same bite force sensor requiring a proportionately larger gape in these smaller animals and potentially reducing bite force performance compared to the larger, dominant naked mole-rats.

### Caveats of the Present Study

*In vivo* measurements from freely behaving animals, as used in the current study, can elicit highly variable responses depending on the motivation level of the animals and how successful the experimental conditions are at eliciting a true maximum bite force. As such, in spite of care being taken to elicit a true maximum bite force, any sub-optimal experimental conditions may underestimate an animal’s actual capabilities. A number of factors can impact biting behaviors. One such variable is ambient temperature, which has been shown to alter biting behaviors in reptiles due to their inability to thermoregulate (Anderson et al., 2008), a quality also characterizing naked mole-rats (Johansen et al., 1976). In addition, *in vivo* bite forces are often elicited as defensive behavioral responses, thus making the effectiveness of experimental conditions in eliciting defensive behaviors a factor in accurately capturing true maximum bite force. Under our experimental conditions, the force sensor probe was combined with a blocked tunnel exit. This was perceived as an agitation and possibly also as a threat, given that our observations indicate that naked mole-rats become aggressive when a tunnel exit is blocked. One role of subordinate animals is that of defenders of the colony, making defensive biting behaviors more likely to be elicited from subordinate animals. Conversely, the dominant naked mole-rats may have been less motivated to bite under such conditions due to their minimal role in defense-related behaviors associated with their higher status in the eusocial hierarchy of the colony (Sherman et al., 1991). Previous studies have shown a positive correlation between defensive biting frequency and body mass, particularly for non-breeding subordinate males that participate in aggressive behaviors such as incisor fencing, biting, shoving, tugging, and open-mouth gaping (Sherman et al., 1991). The lack of an animate threat beyond a blocked tunnel exit could potentially have resulted in diminished defensive biting behaviors elicited in the present study compared to studies in which predators (e.g., snakes) and conspecifics were presented (Sherman et al., 1991). Finally, our comparisons to other mammalian species were weighted toward larger carnivorans for which there are disproportionately more existing data in the literature than for non-predatory species such as ungulates. This over-representation of carnivorans, in addition to differences in the teeth being assessed for bite force calculations (canines, molars, or incisors) and associated gape angle differences as described above, may have skewed our regression analyses (Table 2 and Figure 6). However, naked mole-rats out-performed all carnivorans included in the present analyses, and the addition of ungulates would very likely serve to increase rather than diminish our reported BFQ values for naked mole-rats.

### Bite Force Methodological Differences and Future Directions

Previous studies assessing bite force have relied on a range of different methodologies. These have included: bite force directly measured from freely behaving animals; bite force directly measured in anesthetized animals, elicited via electrical stimulation of masticatory muscles; and bite force values that are predicted based on extrapolations from skull morphology parameters. Variations in BFQs among species may be attributed in part to the methodologies of the studies performed. Across studies, there are differences in how a bite was either directly measured or calculated (annotated as MV for directly measured values or C for calculated values in Table 2 for cross-species comparisons included in the present study). The specific teeth assessed for bite force can also differ (i.e., molars, incisors, or canines), in addition to the environmental conditions and the animal’s motivation to perform, as described above (Thomason et al., 1990; Van Eijden, 1991; Aguirre et al., 2002; Dumont and Herrel, 2003; Thompson et al., 2003; Van Daele et al., 2008; Ellis et al., 2009; Williams et al., 2009). Some bite force studies focused on wild animals that were momentarily restrained within their natural habitat and were (relatively) freely behaving, whereas others assessed wild or laboratory animals in a laboratory setting. In calculated bite force measurements, variations in the specific skull morphology parameters may have resulted in differences in extrapolated bite force predictions (Lucas et al., 1994; Wroe et al., 2005). In the present study, we feel that accurately representative naked mole-rat bite force values were obtained given that the relatively long experimental trials offered ample opportunity for the animals to produce a maximum bite force (five trials of approximately 20 min each, compared to other studies in which five total bites were recorded and the maximum was analyzed, e.g., Van Daele et al., 2008). In addition, our results are consistent with other studies predicting high bite force values for *H. glaber* based on skull morphology and cranial musculature (Cox and Faulkes, 2014; McIntosh and Cox, 2016). Future studies involving electrical stimulation of masticatory muscles, individually and in combination, would be useful in elucidating the extent to which each muscle contributes to overall bite force in naked mole-rats. In addition, intracortical microstimulation (ICMS) electrophysiological experiments targeting primary motor cortex could delineate mototopy and characterize evoked jaw movements that subserve bite force capabilities. Finally, further behavioral studies would be needed to link bite force differences to distinct hierarchical roles (e.g., defender or caretaker) in the naked mole-rat colony. This would be of particular interest in relation to: (1) longitudinal studies focused on specific animals with naturally changing social roles over time, particularly if a subordinate animal transitioned to dominant status, or (2) experimental interventions (e.g., removal of a queen, or removing animals from an established colony to start a new colony) causing shifts in social status that could then be directly related to bite force changes.

## Conclusion

Previous studies have shown that naked mole-rats possess qualities (biting style, skull shape, and masticatory muscle size) indicative of the potential to produce powerful bite forces. This study supported these hypotheses by demonstrating that naked-mole rats produce maximum bite forces that greatly surpass the bite force predicted for their body mass. Subordinate naked mole-rats in particular drive this increase in measured bite force compared to predicted values with their significant, positive allometric relationship of bite force to body mass, biting with forces 65% stronger than the capacity indicated by their body mass based on mammalian cross-species comparisons. This places naked mole-rat bite force performance well above most mammalian species studied to date, surpassing even carnivorans such as lions, wolves, and bears that traditionally epitomize bite strength.

## Data Availability Statement

The datasets generated for this study are available on request to the corresponding author.

## Ethics Statement

All experiments were performed under a protocol approved by the Southern Illinois University Institutional Animal Care and Use Committee and in accordance with the National Institutes of Health Guide for the Care and Use of Laboratory Animals (NIH Publication Nos. 8023 and 1978).

## Author Contributions

DS, BC, and SP contributed to experimental design. NH, CG, and MS collected data for the study. NH, DS, CG, and MS analyzed the data. NH and DS interpreted the data and prepared the manuscript figures. NH and DS drafted and revised the manuscript.

## Conflict of Interest

The authors declare that the research was conducted in the absence of any commercial or financial relationships that could be construed as a potential conflict of interest.

## Supplementary Material

FIGURE S1 Bite force sensor calibration with representative data collection from one experimental trial session and data collection system schematic. **(A)** A calibration curve was generated prior to using each force sensor for data collection, and shows the standardized force (N) applied to a piezo-resistive force sensor (Tekscan; Boston, MA, United States), as well as the resulting force sensor digital input. The representative sensor responded well within the manufacturer’s ± 3% linearity specifications (*R*^2^ = 0.9918). **(B)** Data acquired from a representative bite force experimental session (one trial) is shown for one dominant adult naked mole-rat (female, 1RW, 74 g). Data were sampled at 128 Hz. **(C)** Top left inset of a naked mole-rat biting a force sensor that has been coated in protective Plasti-Dip, along with a schematic of the bite force measurement data collection system. A = 201 Flexiforce sensor (Tekscan; Boston, MA, United States), B = Tekscan Flexiforce Quickstart Board (Tekscan; Boston, MA, United States), C = Adafruit Powerboost 1000C (Adafruit; New York City, NY, United States), D = Adafruit 16bit I2C ADC + PGA ADS1115 (Adafruit; New York City, NY, United States), and E = Raspberry Pi Model B v1.2 (Adafruit; New York City, NY, United States). GND = ground, SCL = serial clock, SDA = serial data, and V = voltage.

FIGURE S2 Illustration of two separate criteria applied in determining bite force frequency. Representative bite force data are shown from a single trial for a dominant adult female naked mole-rat (1RW). **(A)** Inclusive bite frequency criteria: illustration of data points included in the number of bites used to determine bite frequency (gray circles). All points above threshold (at least two standard deviations above the mean) were included to generate the number of bites per trial. This bite frequency was then averaged for each animal across five trials. **(B)** Stringent bite frequency criteria: illustration of data points included in analyses. Red circles represent points above threshold with a minimum of 50 acquired data points between each designated bite event.
