# Supplementary figures and images for: The Better to Eat You With: Bite Force in the Naked Mole-Rat (Heterocephalus glaber) Is Stronger Than Predicted Based on Body Size
Source: Front Integr Neurosci. 2019 Dec 4;13:70. doi: 10.3389/fnint.2019.00070 (PMC6904307; doi:10.3389/fnint.2019.00070)

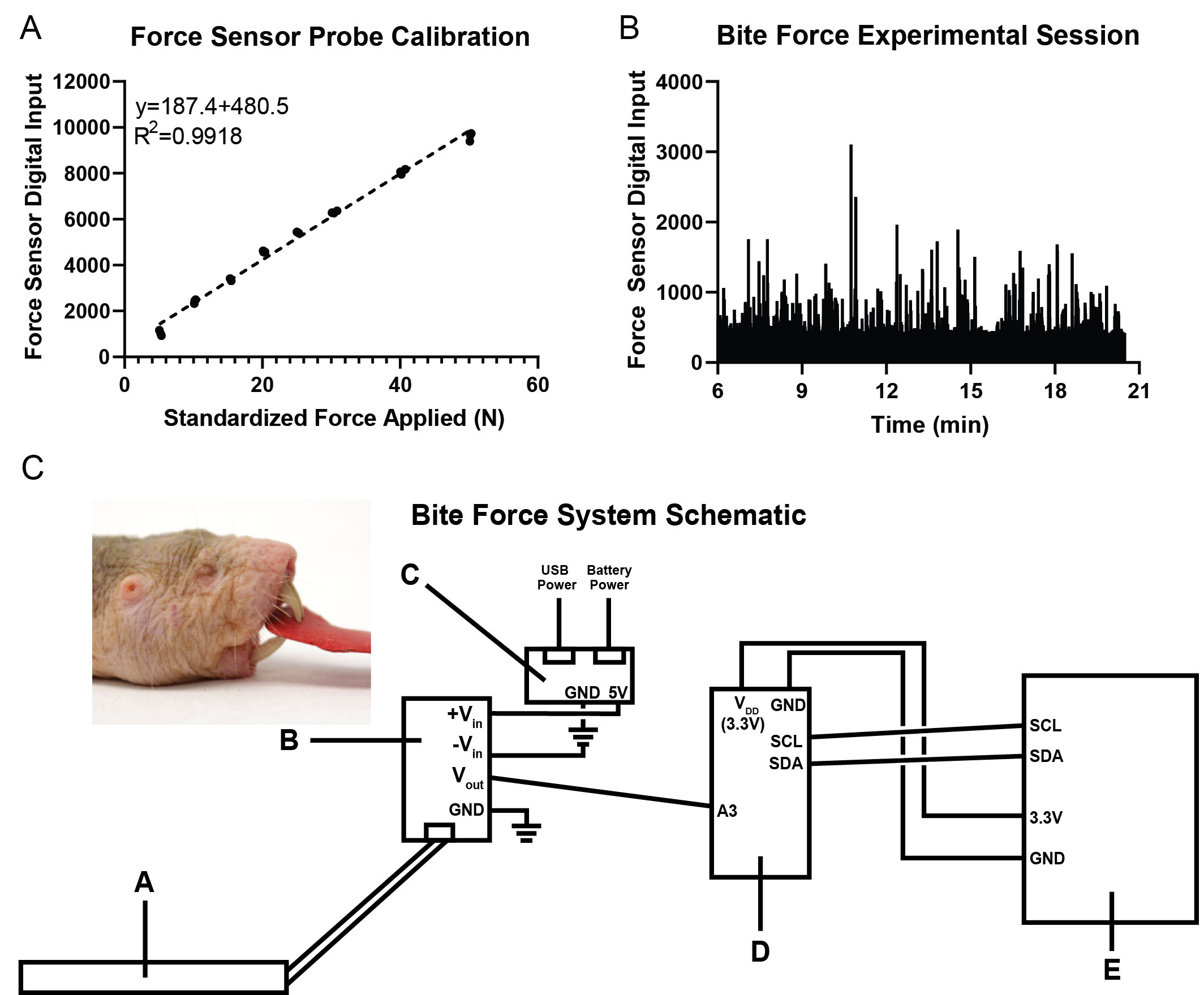

Supplement: FIGURE S1 — Bite force sensor calibration with representative data collection from one experimental trial session and data collection system schematic. (A) A calibration curve was generated prior to using each force sensor for data collection, and shows the standardized force (N) applied to a piezo-resistive force sensor (Tekscan; Boston, MA, United States), as well as the resulting force sensor digital input. The representative sensor responded well within the manufacturer’s ± 3% linearity specifications (R2 = 0.9918). (B) Data acquired from a representative bite force experimental session (one trial) is shown for one dominant adult naked mole-rat (female, 1RW, 74 g). Data were sampled at 128 Hz. (C) Top left inset of a naked mole-rat biting a force sensor that has been coated in protective Plasti-Dip, along with a schematic of the bite force measurement data collection system. A = 201 Flexiforce sensor (Tekscan; Boston, MA, United States), B = Tekscan Flexiforce Quickstart Board (Tekscan; Boston, MA, United States), C = Adafruit Powerboost 1000C (Adafruit; New York City, NY, United States), D = Adafruit 16bit I2C ADC + PGA ADS1115 (Adafruit; New York City, NY, United States), and E = Raspberry Pi Model B v1.2 (Adafruit; New York City, NY, United States). GND = ground, SCL = serial clock, SDA = serial data, and V = voltage. [file Image_1.JPEG]

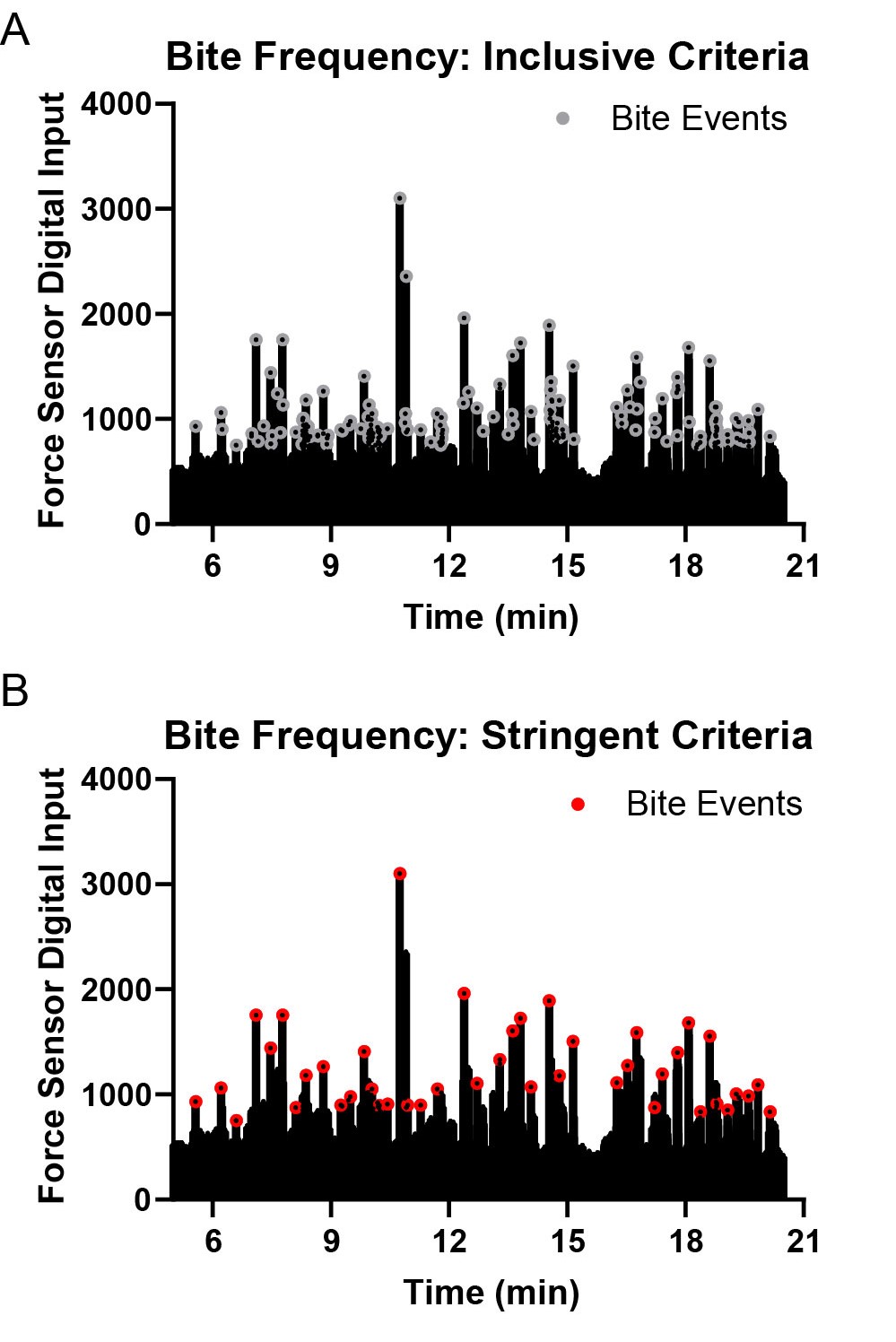

Supplement: FIGURE S2 — Illustration of two separate criteria applied in determining bite force frequency. Representative bite force data are shown from a single trial for a dominant adult female naked mole-rat (1RW). (A) Inclusive bite frequency criteria: illustration of data points included in the number of bites used to determine bite frequency (gray circles). All points above threshold (at least two standard deviations above the mean) were included to generate the number of bites per trial. This bite frequency was then averaged for each animal across five trials. (B) Stringent bite frequency criteria: illustration of data points included in analyses. Red circles represent points above threshold with a minimum of 50 acquired data points between each designated bite event. [file Image_2.JPEG]
